# The quiet flame: whole-body MRI and the underestimated burden of pediatric chronic non-bacterial osteomyelitis (CNO)

**DOI:** 10.1007/s00431-026-07311-9

**Published:** 2026-08-25

**Authors:** Ingrid Galvis, Diego Jaramillo, Lisa F. Imundo, Jorge Delgado, Ola Kvist

**Affiliations:** 1 Department of Radiology, Columbia University Irving Medical Center, New York, NY USA; 2 Department of Pediatric Imaging, Hospital for Special Surgery, New York, NY USA; 3 Department of Pediatrics, Columbia University Irving Medical Center, New York, NY USA; 4 Department of Women’s and Children’s Health, Karolinska Institutet, Stockholm, Sweden

**Keywords:** Chronic non-bacterial osteomyelitis, Pediatric bone disorders, Bone marrow edema, Nonbacterial osteomyelitis

## Abstract

To characterize the radiologic spectrum, lesion distribution, and diagnostic features of Chronic non-bacterial osteomyelitis (CNO) in pediatric patients, including key imaging mimics, and to determine whether clinical presentation correlates with imaging disease burden. Retrospective study included patients younger than 21 years diagnosed with CNO between 2014 and 2024. Clinical presentation, lesion distribution, MRI and radiographic findings, and biopsy results were reviewed. Imaging mimics—including infection, malignancy, and trauma—were evaluated for differential diagnosis. Thirty-two pediatric patients met inclusion criteria (median age 10 years; 62% female). A total of 144 MRI lesions were identified (median 4.5 per patient, range 1–11). Most commonly involved bones were the femur (65.6%), tibia (56.3%), sacrum (31.3%), clavicle (37.5%), and ilium (18.8%). Clinical presentation did not correlate with imaging disease burden: median MRI lesion counts were similar across clinical symptoms (pain: 3.50 lesions; swelling: 5.50; pain and swelling: 1.50; abnormal gait and pain: 4.00; Kruskal–Wallis p = 0.627). This dissociation between clinical symptoms and lesion extent demonstrates that imaging findings cannot be predicted from clinical presentation alone. Whole-body MRI (WB-MRI) performed in 22 patients (68.8%), yielded more lesions (122 lesions, median 5.5 per patient), than regional MRI performed in 10 patients (31.3%) (22 lesions, median 2.0 per patient, p = 0.003).

*Conclusion*: CNO demonstrates multifocal metaphyseal marrow edema with clavicular involvement in most of the cases, distinguishing it from mimics. The pronounced clinical-radiologic dissociation and high lesion burden found at the time of imaging emphasize the occult nature of CNO. These findings highlight the critical need for early diagnostic protocols, such as systematic baseline WB-MRI, to mitigate the substantial diagnostic delays and high lesion burden demonstrated in this cohort.

**Table:** 

**What is Known:** • *Chronic non-bacterial osteomyelitis (CNO) is a rare pediatric autoinflammatory bone disorder characterized by recurrent, sterile multifocal lesions predominantly aff ecting the metaphyses of long bones, with clinical features that frequently overlap with infection and malignancy.*	
**What is New:** • *Whole-body MRI (WB-MRI) detects significantly more lesions per patient than regional MRI, revealing a pronounced dissociation where initial clinical symptoms fail to predict the true extent of occult skeletal disease burden.* • *A 10-year institutional review demonstrates that substantial subclinical lesion burdens accumulate during prolonged diagnostic delays, emphasizing the critical need for early, systematic baseline WB-MRI.*

**What is Known:**

• *Chronic non-bacterial osteomyelitis (CNO) is a rare pediatric autoinflammatory bone disorder characterized by recurrent, sterile multifocal lesions predominantly aff ecting the metaphyses of long bones, with clinical features that frequently overlap with infection and malignancy.*

**What is New:**

• *Whole-body MRI (WB-MRI) detects significantly more lesions per patient than regional MRI, revealing a pronounced dissociation where initial clinical symptoms fail to predict the true extent of occult skeletal disease burden.*

• *A 10-year institutional review demonstrates that substantial subclinical lesion burdens accumulate during prolonged diagnostic delays, emphasizing the critical need for early, systematic baseline WB-MRI.*

## Introduction

First characterized by Giedion et al. in 1972 as subacute and chronic symmetrical osteomyelitis [1], chronic non-bacterial osteomyelitis (CNO) also called chronic recurrent multifocal osteomyelitis (CRMO), is a non-infectious, autoinflammatory bone disorder that primarily affects children and adolescents, with a peak onset around nine years of age and a female-to-male ratio of approximately 2:1 as characterized in large international Eurofever registry [2, 3], presenting across distinct phenotypic patterns of anatomic lesion distribution [4]. It is characterized by recurrent episodes of bone pain and swelling, typically involving multiple sites (multifocal), with periods of exacerbation and remission. The most affected sites are the metaphyses of long bones but the pelvis, spine, clavicle, and mandible can also be involved. Lesions are sterile, with no evidence of bacterial infection, and are often detected as osteolytic or sclerotic changes on radiographs, or areas of marrow edema on MRI, which is the gold standard for diagnosis [5–10].

The pathogenesis is not fully understood but involves immune dysregulation and genetic susceptibility. Current evidence implicates dysregulation of pro- and anti-inflammatory cytokines, leading to chronic bone and soft tissue inflammation. Increased receptor activator of nuclear factor-kB (RANK)–RANK ligand (RANKL) signaling promotes osteoclast differentiation and activation, with tumor necrosis factor-alpha (TNF-a) and interleukin-6 (IL-6) further driving bone resorption and remodeling [11]. Laboratory findings are nonspecific and bone biopsies typically show chronic inflammation without infection [5, 10, 12].

CNO is a diagnosis of exclusion, requiring careful differentiation from infectious osteomyelitis and malignant bone tumors, such as Ewing sarcoma or leukemia [5, 7, 13]. Differentiating CNO from those conditions is critical because clinical and imaging features may overlap, but treatment and prognosis differ significantly. This differentiation is especially important in patients below the age of 3 years as supported by the recently published classification criteria for pediatric CNO [12]. Young patients are more likely to have disease mimickers including hypophosphatasia, infection, malignancy and vitamin D deficiency. Additionally, misdiagnosis may lead to prolonged courses of antibiotics, unnecessary radiation exposure from multiple plain radiographs or bone scans and repeated bone biopsies.

Whole-body MRI (WB-MRI) has become the optimal imaging modality for initial CNO staging because it is more sensitive than bone scintigraphy and radiography. However, a major knowledge gap exists in understanding the degree of association between a patient’s initial clinical symptom and the true extent of occult multifocal bone disease detected by WB-MRI. This study aims to address this issue by comparing disease burden and diagnostic features in a retrospective 10-year institutional pediatric experience and describing the key imaging mimics.

## Methods

This retrospective study was performed at an academic medical center. The study was approved by the institutional review board (Columbia University IRB-AAAV8157) and research activities were in full accordance with all applicable federal and state regulations, including HIPAA privacy. Medical records and imaging studies of pediatric patients diagnosed with CNO between January 2014 and October 2024 were reviewed.

Inclusion criteria were: (1) age under 21 years at time of diagnosis, (2) clinical and radiologic diagnosis of CNO confirmed through retrospective application of the Jansson and Bristol criteria [14] by both pediatric radiologists independently, with disagreements resolved through consensus discussion, and (3) availability of at least one MRI and/or radiographic study for review. If a patient had more than one MRI, only the first MRI was selected. Exclusion criteria were: final diagnosis of infectious osteomyelitis, neoplastic bone lesion, trauma, or insufficient imaging or clinical documentation. Imaging studies were analyzed in consensus by two pediatric radiologists (O.K. and J.D, with over 5 years of experience in pediatric radiology, respectively). The radiologists were blinded to the clinical findings.

Imaging was performed according to routine WB-MRI clinical protocols at our institution. This protocol varied over time, but consisted in a combination of axial and coronal STIR, coronal T1, axial T1, axial T2-weighted, and sagittal STIR imaging of the spine. The regional imaging (typically included coronal and sagittal T1-weighted, T2-weighted, and STIR or fat-suppressed sequences, with post-contrast T1-weighted imaging) when indicated. Specific field strength data (1.5 T vs 3 T) and standardized protocol parameters were not systematically documented in the retrospective record review.

### Statistical analysis

Differences in total lesion counts across symptom groups were assessed using the Kruskal–Wallis H test, A generalized linear model with negative binomial distribution was additionally fitted to account for overdispersion. Per-patient lesion counts between whole-body and regional MRI were compared using the Wilcoxon rank-sum test, while total lesion distribution by imaging type was assessed using chi-square test. Spearman rank correlation was used to assess the association between the number of symptomatic clinical locations per patient and total MRI lesion count. All statistical analyses were performed using SAS, version 9.4 (SAS Institute Inc., Cary, NC, USA), and R, version 4.5.1 (R Foundation for Statistical Computing, Vienna, Austria), and p value < 0.05 was considered statistically significant.

## Results

### Patient demographics and clinical presentation

A total of 32 pediatric patients met inclusion criteria for CNO over the 10-year period. Descriptive statistics were reported as numbers with percentages or medians with interquartile ranges. The mean age at symptom onset was 10.2 years (± 3.6,3–21). The sample included 20 females (62%) and 12 males (38%). The most common presenting symptoms were antalgic gait (n = 8; 25%), hip pain (n = 7; 22%), and clavicle pain/swelling (n = 7; 22%), and knee pain (n = 5;16%). 10 patients (31%) presented more than one symptom at the time of diagnosis. The locations of the reported symptoms were hip (n = 9; 28.1%), clavicle (n = 7;21.9%), knee (n = 6; 18.8%), ankle (n = 5; 15.6%), and leg (n = 5; 15.6%). 1 patient presented with systemic signs of infection such as fever and elevated inflammatory markers at diagnosis. The length of time between clinical exam and diagnosis varied substantially, 6 patients experienced delays, with intervals ranging from 2 to 9 years (mean = 3.8 years). Most of these cases had other rheumatologic diseases or diagnosis was confused with osteomyelitis. All included patients received treatment with non-steroidal anti-inflammatory drugs (NSAID), sometimes for many months before the MR examination took place. Patient characteristics are displayed in Table 1 (see Table 1).

**Table 1:** Patient Characteristics and Clinical Presentation (32 patients)

Variables	Value
**Demographics**	
**Sex, F:M (ratio)**	20:12 (5:3)
**Age at diagnosis, years, mean ± SD (range)**	10.7 ± 4.1(3–21)
**Age at onset, years, mean ± SD (range)**	10.2 ± 3.6(3–21)
**Delay in diagnosis, years, mean (range)**	3.8 (2–9 years)
**Medical History of Autoimmune disease**	10 (31.2%)
**Symptoms**	
**Localized pain**	24 (75%)
**Local inflammation (swelling)**	10 (31.3%)
**Abnormal gait**	8 (25%)
**Fever/fatigue**	1 (3.1%)
**Polyarthralgia**	1 (3.1%)
**Ezcema**	1 (3.1%)
**Clinical locations per patient, Median (range)**	1 (1–4)
**Symptomatic sites**	
Hip	9 (28.1%)
Clavicle	7 (21.9%)
Knee	6 (18.8%)
Ankle	5 (15.6%)
Leg	5 (15.6%)
Elbow	1 (3.1%)
Shoulder	1 (3.1%)
Foot (metatarsals)	1 (3.1%)
Jaw	1 (3.1%)
**Total number of MRI lesions**	144
**MRI lesions per patient, Median (IQR)**	4.5 (4.25)

## MRI findings and lesion distribution

MRI protocols varied over the 10-year study period due to equipment upgrades and evolving clinical practices. Twenty-two patients underwent whole-body MRI while ten had regional MRI targeted to symptomatic areas, based on clinical indication and physician preference. All patients consistently demonstrated characteristic features of CNO. The most prominent finding was multifocal bone marrow edema, predominantly centered in the metaphyseal regions of long bones, with occasional extension into the epiphyses, followed by periosteal reaction and osteitis. Lesions exhibited low signal intensity on T1-weighted images and high signal intensity on STIR sequences, reflecting active inflammation. Importantly, there was a complete absence of intraosseous abscess formation, cortical bone destruction, or associated soft tissue masses, helping to differentiate CNO from infectious osteomyelitis or neoplastic processes. Post-contrast imaging revealed intense enhancement of the marrow and, in some cases, surrounding soft tissues—supportive of inflammatory etiology. No aggressive imaging features such as sequestrum formation, sinus tract development, or periosteal lifting were observed. The imaging pattern was highly suggestive of a non-infectious, inflammatory etiology.

**Fig. 1:**
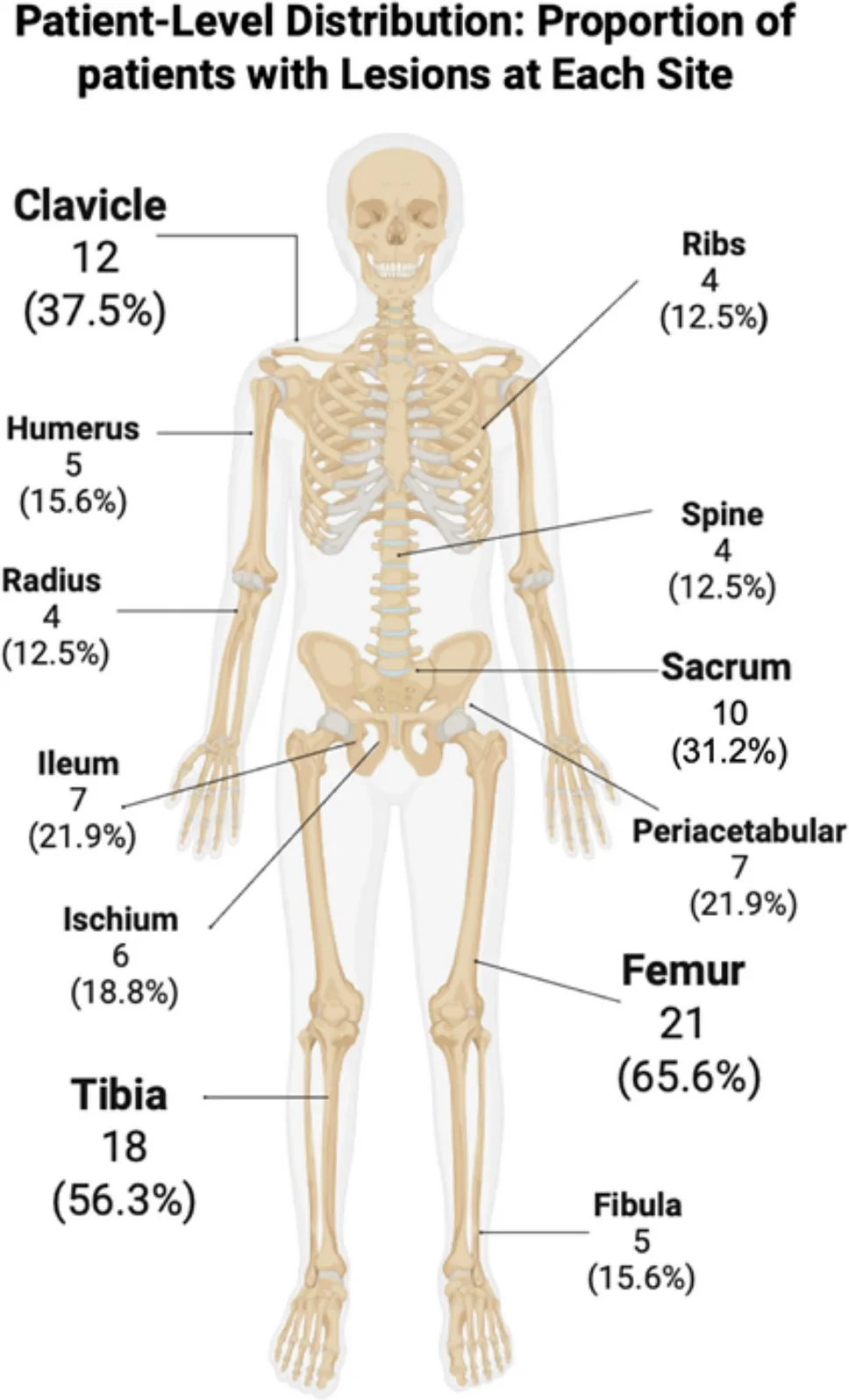
Patient-Level Distribution: Proportion of Patients with Lesions at Each Site (*n* = 32). Values indicate the number and percentage of patients with at least one lesion at each site: Femur 21 (65.6%), Tibia 18 (56.3%), Sacrum 10 (31.2%), Clavicle 12 (37.5%), Periacetabular 7 (21.9%), Ischium 6 (18.8%), Thoracic/Lumbar-spine 4 (12.5%), Ileum 7 (21.9%), Humerus 5 (15.6%), Fibula 5(15.6%), Ribs 4(12.5%), Radius 4 (12.5%)

A total of 144 MRI lesions were identified across the study sample, with a median of 4.5 per patient. The appendicular skeleton was the most involved with femoral and tibial involvement predominating, whereas the sacrum and vertebrae were the most frequently affected axial sites (Fig. 1). Overall, the lesion distribution demonstrated a predilection for weight-bearing bones. The majority of lesions (117 of 144, 81.2%) occurred in the 5–9 and 10–12-year groups, while older adolescents (16–18 and 19–21 years) had relatively fewer findings (15 of 144, 10.4%). Median MRI lesion counts by symptom group were: pain 3.50 (IQR 2.00–6.00), swelling 5.50 (IQR 3.00–6.50), pain + swelling 1.50 (IQR 1.00–10.00), and abnormal gait + pain 4.00 (IQR 4.00–7.00). The Kruskal–Wallis test revealed no significant overall difference in lesion counts across symptom groups (χ^2^ = 1.75, df = 3, p = 0.627). Because lesion counts were overdispersed, a negative binomial regression model was additionally used to confirm results. It similarly demonstrated no significant differences when comparing each symptom group to the reference category (all p > 0.39). This finding indicates that clinical presentation does not reliably reflect true disease burden.

Over the study period, 22 patients (68.8%) underwent whole-body MRI (WB-MRI) and 10 patients (31.3%) targeted regional MRI based on clinical indication and physician preference. When normalized by per-patient lesion counts, WB-MRI patients had a significantly higher median of 5.5 lesions (mean = 5.55 ± 3.13, IQR 3.0–7.0, range 1–11) compared to 2.0 lesions in regional MRI patients (mean = 2.2 ± 1.32, IQR 1.0–4.0, range 1–4). This difference was statistically significant (Wilcoxon rank-sum test, Z = − 2.98, p = 0.003), indicating that WB-MRI detected 2.75 times more lesions per patient. In absolute terms, WB-MRI identified 122 lesions (84.7% of total) compared to 22 lesions (15.3% of total) with regional MRI, χ^2^ = 69.44, p < 0.0001. This substantial difference reflects both the superior sensitivity of WB-MRI for detecting inflammatory lesions and its comprehensive anatomic coverage of the entire skeleton. Additionally, the number of symptomatic clinical locations per patient showed no correlation with total MRI lesion count (Spearman ρ = − 0.03, p = 0.879), further confirming that disease extent on imaging cannot be predicted from the distribution of clinical symptoms.

## Biopsy findings

Image-guided bone biopsy was performed in 13 patients (41%) with persistent symptoms despite conservative management, when there was diagnostic uncertainty, or when infection or malignancy could not be confidently excluded. Histopathologic analysis consistently demonstrated reactive woven bone, fibromyxoid marrow, and occasional focal necrosis across all biopsied cases. No evidence of malignant cells or granulomatous inflammation. All cultures were negative, reinforcing the non-infectious etiology.

## Discussion

The most clinically significant finding of this 10-year institutional study is the dissociation between the patient’s initial clinical presentation and the true extent of skeletal disease burden as quantified by Whole-Body MRI (WB-MRI). Anchoring our demographic and epidemiological parameters within the benchmark established by foundational early CNO cohorts [1, 2, 14], our study builds on this foundation by analyzing a robust 10-year cohort to look closer at the exact nature of this clinical-imaging mismatch. While CNO is recognized for its multifocal nature, our analysis demonstrated that median WB-MRI lesion counts were statistically similar across all four clinical symptom groups (Kruskal–Wallis p = 0.627). The pronounced discrepancy between localized clinical symptoms and total lesion burden underscores the highly occult nature of CNO marrow involvement. This finding firmly aligns with an established, two-decade body of literature demonstrating that clinical symptoms severely underestimate total skeletal disease burden, while emphasizing the critical role of whole-body imaging accurate disease staging. Because active bone marrow edema can accumulate within the medullary cavity without immediately causing periosteal stretching or adjacent soft-tissue inflammation severe enough to trigger localized nociception, extensive multi-site disease can develop silently. The clinical-imaging mismatch observed in our cohort is not merely a reflection of WB-MRI’s superior sensitivity, but rather a reflection of regional anatomy. Deep-seated structures like the pelvic girdle and the dorsal elements of the spine can harbor significant osteolytic or inflammatory changes before manifesting as localized physical pain or functional limitations. This explains why patients undergoing WB-MRI demonstrated a significantly higher median lesion count (5.5) compared to those evaluated with regional imaging (2.0, p = 0.003); regional protocols inherently miss clinically silent but anatomically active sites of disease. A patient presenting with seemingly unifocal pain was just as likely to harbor numerous clinically silent lesions as a patient with more complex, multifocal symptoms. This data provides robust evidence that imaging findings cannot be reliably predicted from clinical symptoms alone.

This 10-year institutional study adds to the literature on pediatric CNO by systematically characterizing lesion distribution, correlating imaging findings with histopathology, and evaluating imaging mimics, including infection, malignancy, and trauma. In our study sample, clinical symptoms often localized to sites of MRI-detected lesions, but imaging consistently revealed a broader distribution. Antalgic gait and hip pain corresponded to periacetabular, proximal femoral or sacral marrow lesions, while clavicular pain and swelling strongly correlated with clavicular marrow edema and cortical thickening. Similarly, knee pain was frequently associated with femoral or tibial metaphyseal lesions.

**Fig. 2:**
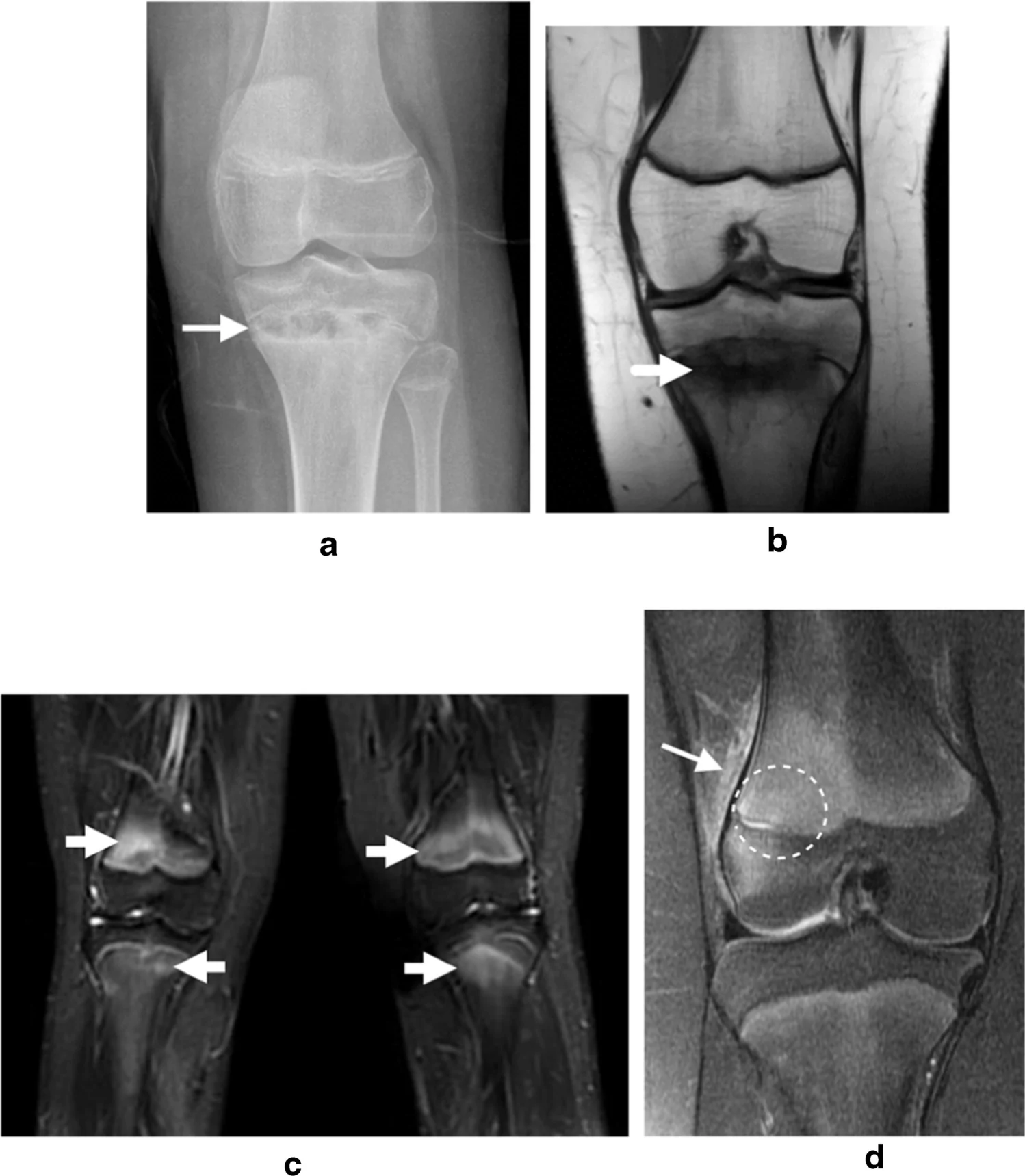
CNO involving the lower extremities. **a** 10-year-old girl with CNO involving the left leg. Anteroposterior radiograph shows mixed lytic and sclerotic changes in the proximal tibial metaphysis (arrow). **b** Coronal T1-weighted turbo spin-echo (TSE) MRI demonstrates low marrow signal extending from the proximal tibial metaphysis into the epiphysis with intact physis (arrow). **c** 3-year-old with extensive workup including biopsy with cytology and culture negative for osteomyelitis, blood cultures, Lyme antibodies, QuantiFERON test for tuberculosis, HLA B27 testing, extensive autoimmune disease evaluation (ANA, RF, Cyclic citrulline peptide IGG among other test) and Vitamin D levels. Coronal T2 STIR MR image of both knees shows areas of hyperintensity involving both distal femoral, and proximal tibial metaphysis (arrows), suggesting CNO. **d** 11-year-old boy with acute, uncomplicated osteomyelitis of the lateral distal metaphysis and epiphysis of the right femur. Coronal T2 fat-suppressed MR image demonstrates mild physeal widening with hyperintense edema in the lateral distal femur (dashed circle), overlying periostitis, and adjacent soft tissue edema (arrow). Trace joint fluid is present without significant inflammatory changes to suggest septic arthritis

We found an inconsistency between the number of clinical locations per patient (median 1.0, range 1–4) and total of MRI lesions (median 4.5, IQR = 4.25). Although radiographs remain a common first-line tool for the evaluation of pediatric bone pain, CNO lesions are frequently radiographically occult. Radiographs detected lesions in only 25% of cases in patients with localized symptoms, substantially lower than MRI, which identified 100% of clinically symptomatic sites and additional subclinical lesions. This gap in sensitivity is consistent with prior reports that emphasize the limited utility of radiographs in CNO. In some cases radiographs may depict characteristic features such as chronic sclerosis and hyperostosis. In contrast, periosteal reaction represents a distinct radiological marker of acute, active cortical inflammation rather than late-stage structural remodeling, occurring typically in metaphyseal regions of long bones and the clavicle, pelvis, and spine (Fig. 2) [7].

The reliance on initial radiographs, combined with the 10-year retrospective span of our study (2014–2024), directly contributes to the relatively prolonged timeline to final diagnosis observed in our cohort. In the earlier half of our study window, clinical awareness of CNO was lower, and the request access to dedicated pediatric WB-MRI was limited. This often resulted in sequential, low-yield regional imaging that delayed definitive characterization [15]. Furthermore, the clinical presentation of CNO frequently mimics more prevalent pediatric conditions such as transient synovitis, growing pains, or low-grade pyogenic osteomyelitis [16, 17]. This diagnostic challenge is particularly pronounced in the youngest patients within our cohort; while older children could articulate localized bone pain, very young children frequently presented with non-specific symptoms such as an unexplained limp or general irritability [16, 18]. This age-dependent presentation often directed initial medical workups toward mechanical or infectious etiologies, further compounding the diagnostic delay and allowing a higher burden of subclinical lesions to quietly accumulate across the skeleton before the diagnosis was finalized.

**Fig. 3:**
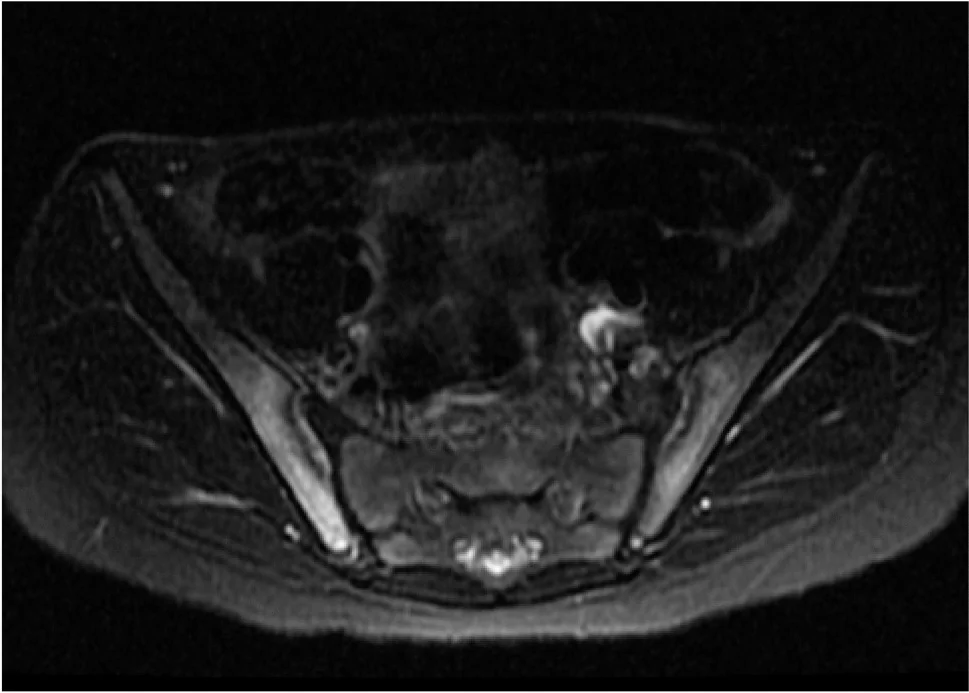
Axial STIR image of a 10-year-old female demonstrates bone marrow edema (abnormal high signal intensity) in bilateral iliac bones adjacent to the sacroiliac joints

**Fig. 4:**
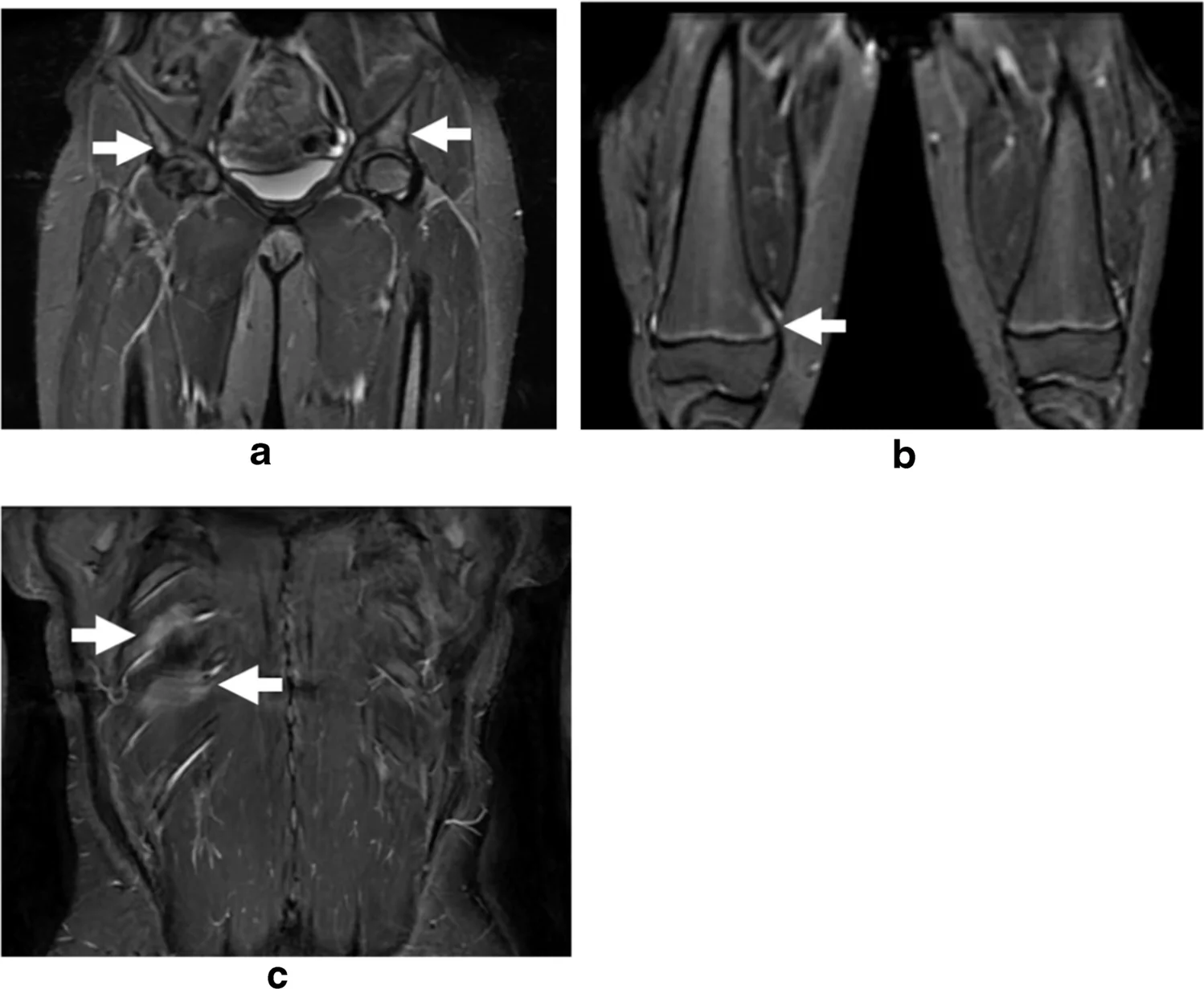
Images obtained in a 12-year-old girl with CNO involving both supra-acetabular regions, right femur, and ribs. **a** Coronal STIR MR image of the hips shows areas of increased signal intensity in both supra-acetabular regions (arrows). **b** Coronal STIR MR image of the knees shows hyperintense areas in the medial metaphysis of the distal right femur (arrow). **c** Coronal STIR MR image of the chest shows patchy T2 signal along the right posterior ninth and tenth ribs (arrows)

Lesions in our study sample showed the typical distribution, predominantly long bone metaphyses, often multifocal and sometimes symmetric lesions, aligning with landmark original imaging series [4, 19]. Long bone metaphyses were the most frequent sites, followed by the clavicle, pelvis, and spine. Clavicular lesions, present in 37.5% of our patients, are a valuable diagnostic clue, as they are rare in other pediatric bone disorders. MRI is widely regarded as the preferred imaging modality for further characterization of lesions due to its superior sensitivity in detecting early inflammatory changes, subclinical lesions, mapping disease extent, and characterizing the typical bilateral, symmetric distribution. MRI images provide distinctive features including cortical thickening and reactive bone edema, with lesions typically appearing hyperintense on STIR or T2-weighted images and hypointense on T1-weighted images, frequently accompanied by periosteal reaction or soft-tissue inflammation (Fig. 3). Crucially, this periosteal response is a classic feature of active, acute-phase CNO bone inflammation rather than a late sequela; it can range from subtle to prominent and occasionally mimic more aggressive processes (Fig. 4) [19].

A particularly striking finding in this cohort was the magnitude of diagnostic delay observed in a subset of patients. Six patients experienced delays ranging from 2 to 9 years (mean 3.8 years) between symptom onset and confirmed CNO diagnosis, which is two to three times longer than the 12–18 months reported in prior series [8]. This discrepancy suggests differences in symptom patterns and initial diagnostic workup in our population. Several factors likely contributed to this prolonged interval. First, 31% of our patients carried a concurrent autoimmune diagnosis, which may have anchored clinical attention to an established rheumatologic framework and deferred consideration of CNO as a distinct entity. Second, initial symptoms in most delayed cases were attributed to infectious osteomyelitis, resulting in repeated antibiotic courses and reliance on radiographs before CNO was considered. Third, within this North American cohort, structural healthcare barriers uniquely compounded these timelines; obtaining insurance pre-authorization for advanced, multi-regional modalities like pediatric WB-MRI frequently introduces extensive administrative delays, forcing clinicians to rely on lower-yield, localized regional imaging or serial radiographs while awaiting approval. Fourth, the 10-year span of this study captures a period of evolving clinical awareness: Roderick et al. demonstrated a statistically significant decreasing trend in time to diagnosis over an 8-year period (R = − 0.29, p = 0.03), attributing this improvement to a targeted regional awareness campaign directed at orthopedic surgeons [15]; cases diagnosed in the earlier years of our cohort likely reflect a lower baseline index of clinical suspicion at our institution before CNO became more broadly recognized.

The poor correlation between clinical symptomatology and total MRI lesion burden underscores the complexity of disease surveillance in this population [19, 20]. While the existence of this clinical-radiological mismatch is well-recognized globally, our single-institution retrospective 10-year experience allowed us to evaluate the exact nature of this divergence under uniform contemporary imaging protocols. This institutional data demonstrates that even presenting symptoms classically considered 'localized' fail to reliably predict or limit the extent of underlying multi-site involvement. However, the utility of systematic, asymptomatic screening via whole-body MRI (WB-MRI) remains limited by challenges in specificity. Recent normative data have demonstrated that focal bone marrow signal intensities simulating active pathology are a frequent, non-specific finding on WB-MRI in healthy, asymptomatic children and adolescents [21, 22]. Consequently, imaging findings must be interpreted with strict clinical and geographic correlation to avoid diagnostic over-escalation. The pronounced lesion burden documented at the time of eventual WB-MRI in this cohort, accumulated silently over years of unrecognized disease, underscores that diagnostic delay may be the principal driver of high disease burden at presentation in this population. Future prospective studies should examine whether earlier specialist referral, particularly in children with recurrent bone pain not explained by infection or trauma, can reduce the interval from symptom onset to confirmed diagnosis.

Our findings demonstrate that WB-MRI could significantly improve detection of CNO lesions compared to targeted regional MRI. Specifically, WB-MRI patients had a median of 5.5 lesions versus 2.0 with regional imaging (p = 0.003), a 2.75-fold difference. This represents an advantage in detecting multifocal disease rather than a protocol artifact reflecting greater anatomic coverage. WB-MRI identified subclinical clavicular, spinal and pelvic involvement in patients who presented with localized symptoms (e.g., isolated clavicular or hip pain), findings that often require treatment escalation and intensive immunosuppression [19, 23–26]. Early and accurate diagnosis not only guides appropriate therapy but may reduce the risk of deformity, chronic pain, and functional limitations [9, 24]. In our study sample, two patients developed lower limb length discrepancy, likely due to prolonged metaphyseal inflammation adjacent to the physis. While vertebral lesions can progress to collapse in untreated CNO, which can lead to permanent loss of vertebral body height in up to 48% of patients with spinal lesions [27].

Kieninger et al. [24] demonstrated that WB-MRI performed within six months of initial presentation resulted in CNO diagnosis within that same six-month period in all cases, whereas delayed WB-MRI extended the diagnostic interval sixfold [24]. These findings from Kieninger et al. highlight what our cohort illustrates from the opposite direction, namely that the lesion burden accumulates when WB-MRI is not deployed early. Beyond improving early detection, it may allow more accurate assessment of disease burden and treatment response, guiding both clinical management and follow-up strategies. Occult lesions detected on imaging can be clinically significant, as involvement of sites such as the axial skeleton often requires treatment escalation (Fig. 4) [19, 23–26]. Additionally, evidence suggests the therapeutic efficacy of nonsteroidal anti-inflammatory medicines (NSAIDs) may be influenced by the duration of active disease [24].

Although composite MRI scoring systems such as the Radiological Index for Nonbacterial Osteitis (RINBO) and Chronic Nonbacterial Osteomyelitis MRI Scoring (CROMRIS) system have been developed to quantify disease activity, their clinical utility remains limited by the lack of validation in large cohorts [20, 28]. CROMRIS provides a structured approach incorporating bone marrow signal intensity, periostitis, soft tissue edema, and spinal involvement, offering potential standardization for longitudinal disease monitoring. However, consistent application across centers is still challenging due to variability in MRI protocols and reader subjectivity.

### Differential diagnoses

While the literature extensively documents where CNO lesions typically appear, the primary clinical challenge remains the differentiation of these lesions from high-stakes mimics. The unique contribution of our series lies in providing a direct, comparative radiological blueprint to distinguish CNO from acute osteomyelitis, hematologic malignancies, and stress injuries. Because the baseline narrative surrounding subclinical CNO distribution is mature, a core objective and strength of this study lies in refining the practical, diagnostic differentiation of key imaging mimics. The imaging appearance of CNO often overlaps with a spectrum of infectious and neoplastic entities, as shown in Table 2. Our study underscores the essential role of whole-body magnetic resonance imaging (WB-MRI) in overcoming this diagnostic challenge, particularly given the observed poor correlation between clinical symptom severity and radiographic extent of disease. On MRI, the principal differential diagnoses include infectious osteomyelitis, malignant bone tumors (particularly Ewing sarcoma and leukemia), Langerhans cell histiocytosis, benign bone tumors such as osteoid osteoma, metabolic bone diseases, and other autoinflammatory or rheumatologic disorders, including juvenile idiopathic arthritis (JIA) [16, 23, 25, 29]. Likewise, juvenile ankylosing spondylitis (JAS) typically affects entheses, sacroiliac joints, and the axial skeleton. However, in long bones, metaphysis involvement is uncommon.

**Table 2:** MRI features of CNO and common differential diagnoses

Feature	CNO	Infectious osteomyelitis	Malignancy (Ewing sarcoma, leukemia)	Avascular necrosis (AVN)
Lesion distribution	Multifocal, often symmetric; metaphyseal and clavicular predilection	Usually unifocal; metaphyseal or diaphyseal	Often unifocal or diffuse (leukemia); diaphyseal or metaphyseal	Subchondral, epiphyseal, focal
T1-weighted signal	Hypointense	Hypointense	Hypointense, diffuse or patchy (especially leukemia)	Hypointense with subchondral band-like low signal
STIR/T2 signal	Hyperintense (marrow edema)	Hyperintense (marrow edema)	Hyperintense (infiltration, mass)	“Double line sign” (inner bright and outer dark line) on T2
Enhancement pattern	Homogeneous marrow enhancement	Irregular; abscess or sinus tract formation	Heterogeneous enhancement; soft tissue mass	Variable; early edema with subchondral enhancement
Periosteal reaction	Variable; typically absent or mild, but can be prominent and occasionally exhibit lamellated/aggressive-appearing morphology	Prominent, irregular	Aggressive or laminated	Typically, absent
Soft tissue changes	Mild or none	Soft tissue abscess or phlegmon	Soft tissue mass (Ewing sarcoma); none (leukemia)	Minimal, joint effusion possible
Key differentiators	Clavicular involvement, absence of abscess or cortical destruction	Abscess, cortical disruption, systemic symptoms	Aggressive periosteal reaction, mass, diffuse marrow replacement	Subchondral collapse or fragmentation in advanced stages

### Acute hematogenous osteomyelitis (AHO)and infectious mimics

**Fig. 5:**
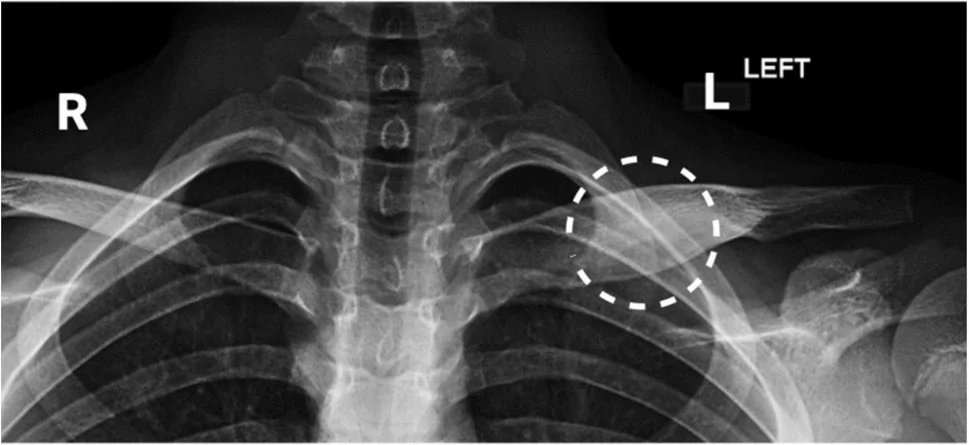
Image obtained in a 9-year-old boy with CNO involving left clavicle. Anteroposterior radiograph shows expansion of the left medial clavicle with mixed sclerotic and lytic changes (dashed circle)

**Fig. 6:**
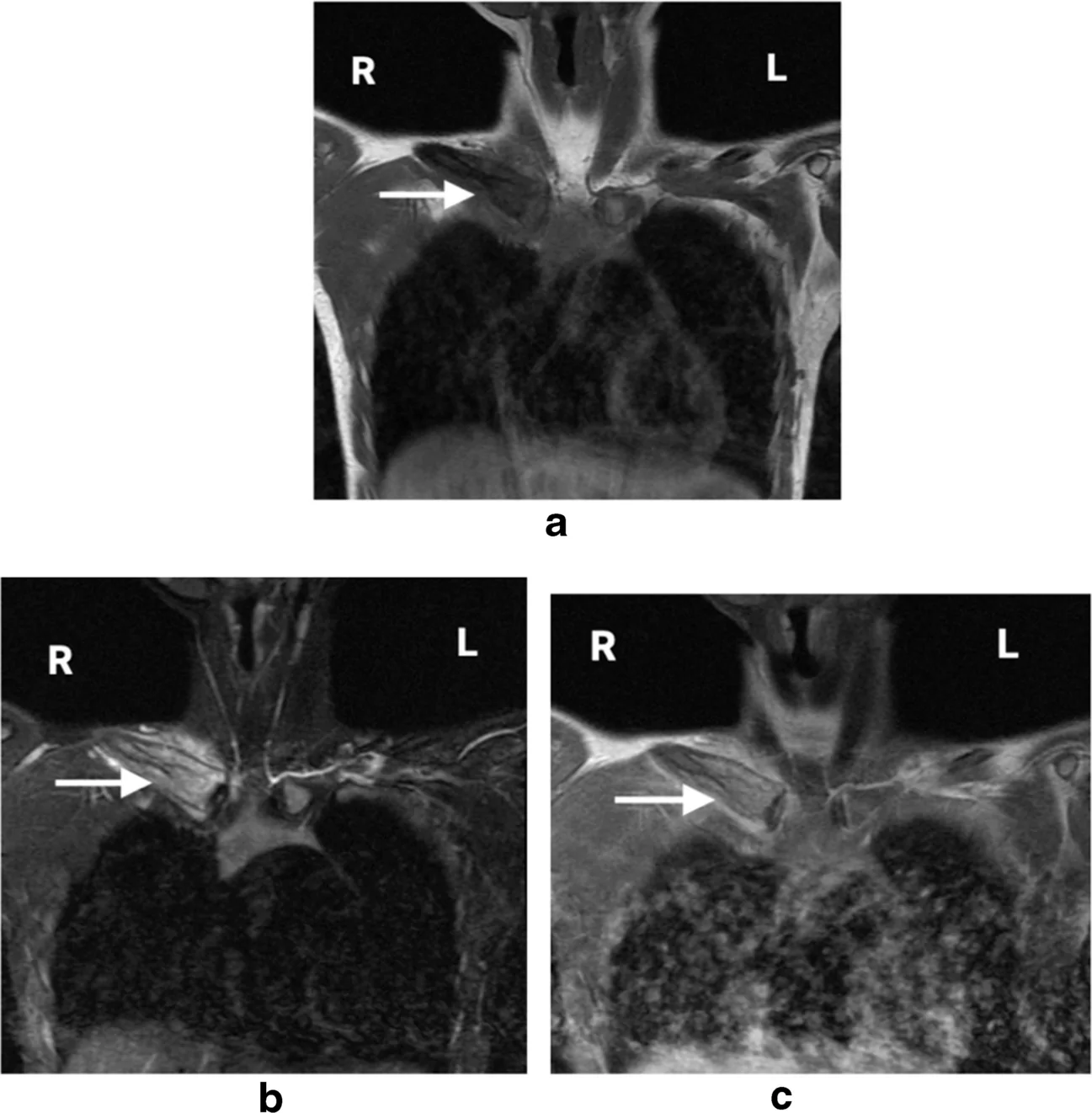
Images obtained of 10-year-old girl with CNO involving the right clavicle. **a** Coronal T1 weighted MR imaging of the right clavicle shows low signal intensity in the medial 2/3 of the right clavicle, extending laterally from the physis on T1 weighted images (arrow), with associated periosteal reaction that may appear prominent or aggressive. **b** Coronal STIR MR imaging of the right clavicle shows high signal intensity (arrow) (**c**) and enhances with gadolinium (arrow)

Distinguishing CNO from bacterial AHO remains the most immediate diagnostic hurdle. While AHO typically presents with a clear clinical picture of fever, focal pain, and pronounced systemic inflammation (elevated C-reactive protein and erythrocyte sedimentation rate), CNO can present with low-grade fever and mildly elevated inflammatory markers, or even be completely asymptomatic, as seen in silent lesions detected by WB-MRI. The gold standard for AHO diagnosis involves positive blood or bone aspirate cultures. Conversely, CNO is a diagnosis of exclusion, supported by sterile cultures and characteristic radiologic patterns. Specifically, the presence of multiple, non-contiguous lesions—often involving the clavicle, metaphyses of long bones, and axial skeleton (sacrum and ilium)—is highly suggestive of CNO and argues strongly against a unifocal infectious process (Fig. 5 and 6). WB-MRI is paramount here, as it can unveil asymptomatic multifocality that strongly favors the diagnosis of CNO [30–32].

### Malignancy (primary and metastatic bone tumors)

**Fig. 7:**
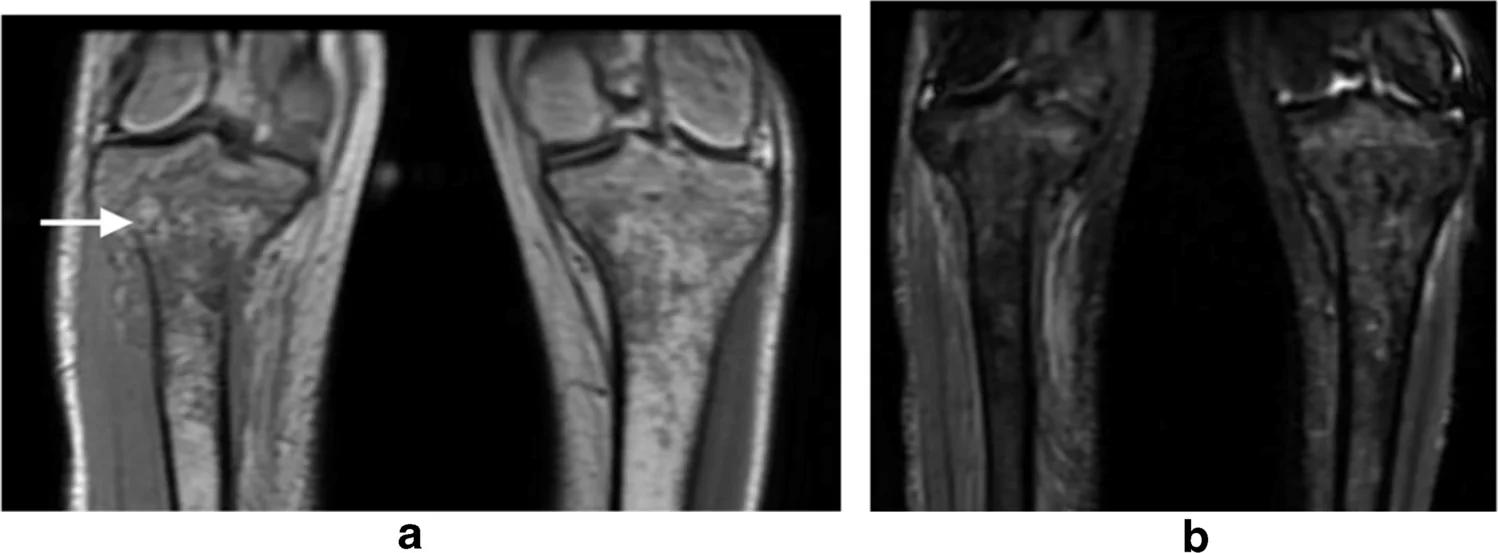
Image obtained in 20-year-old man with acute lymphoblastic leukemia involving both tibias. **a** Coronal T1 MR imaging of the knees shows diffuse heterogeneity of the bone marrow within the bilateral proximal tibias, as well as within the tibias bilaterally, manifested as areas of low T1 signal (solid arrow). **b** Coronal T2 MR imaging of the knees shows patchy areas of high T2 signal. These findings likely represent marrow infiltrative changes associated with leukemia and possible superimposed bone infarcts

Neoplastic lesions, including Ewing sarcoma, osteosarcoma, and metastatic neuroblastoma, must be excluded due to their aggressive nature. WB-MRI offers crucial morphological clues for distinguishing CNO from malignancy. Malignant lesions often display large, destructive soft-tissue masses, aggressive periosteal reaction (e.g., Sunburst or Codman triangles), and rapid progression, which are typically absent in CNO [23, 26, 33]. Among these, acute lymphoblastic leukemia (ALL) warrants special consideration, particularly in cases presenting with diffuse bone marrow signal changes [34–36] (Fig. 7). While CNO lesions can occasionally appear aggressive or unifocal—a known pitfall—they typically lack the extensive cortical destruction and large soft-tissue component characteristic of high-grade sarcomas [37–39]. Furthermore, the inherent symmetry and predilection for certain sites (e.g., the clavicle and sacroiliac joints) in CNO are seldom seen in pediatric bone malignancies. In cases of unifocal CNO where imaging is equivocal, biopsy remains essential, revealing the characteristic sterile, plasma cell-rich inflammatory infiltrate of CNO rather than malignant cells.

### Traumatic lesions

**Fig. 8:**
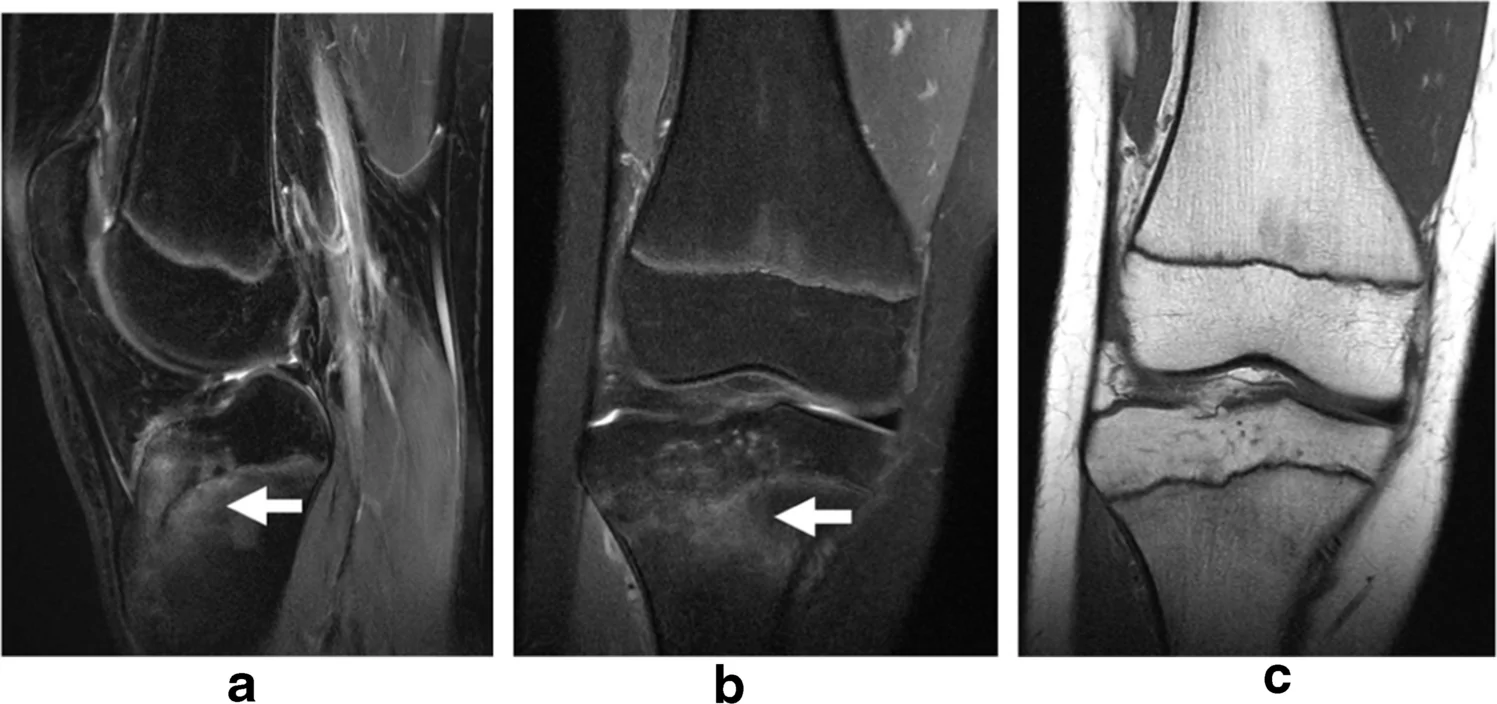
Imaged obtained in 17-year-old boy who presented with pain in the right knee following a contusion. **a** Sagittal T2 FS MR image shows mild hyperintense signal within the posterior aspect of the proximal tibial metaphysis consistent with mild bone marrow edema (arrow). **b** Coronal PD FS MR image of the left knee shows patchy areas of hyperintense signal in the metaphyseal region of the proximal tibia (arrow). **c** Coronal T1MR image shows normal T1 marrow signal with intact cortex and preserved cartilage of the proximal tibia

A frequent yet often overlooked differential in athletic pediatric patients is bone contusions, which manifest on MRI as focal bone marrow edema, with low signal intensity on T1-weighted images and high signal on T2/STIR sequences, typically without cortical break or associated soft tissue mass [39]. These lesions are usually centered near the site of impact and tend to resolve over time (Fig. 8). Another diagnostic challenge is that a high proportion of healthy, asymptomatic children and adolescents may exhibit bone marrow signal abnormalities in the long bones of the lower extremities and axial skeleton [21, 22, 40]. These focal or patchy hyperintensities on T₂-weighted and fat-suppressed images are typically symmetric, bilateral, and without associated periosteal or soft tissue involvement [40]. Awareness of this is important when interpreting whole-body MRI in this age group, particularly in the assessment of clinically silent lesions to avoid overdiagnosis of CNO and prevent unnecessary interventions.

### Advanced MRI techniques

Diffusion-weighted imaging (DWI) with apparent diffusion coefficient (ADC) mapping has been used as an adjunct to standard whole-body STIR/T1 protocols to provide additional tissue characterization, even if it does not detect more lesions than STIR [19, 33, 41]. ADC has not yet been validated as a standalone biomarker of clinical severity or therapeutic response in CNO but the values in lesions are typically elevated compared to adjacent normal marrow, reflecting increased free water content from inflammatory edema rather than restricted diffusion from high cellularity [19, 20]. This pattern can support a benign inflammatory diagnosis over malignancy when the differential is uncertain, and potentially reduce unnecessary biopsies. DWI can enhance diagnostic accuracy, its absence in our protocol likely did not affect lesion count but may have limited lesion characterization.

### Implications for practice

The imaging findings of CNO have direct clinical implications. Recognition of characteristic imaging features, namely multifocal metaphyseal marrow edema, clavicular predilection, and absence of aggressive findings, may facilitate earlier diagnosis and reduce reliance on unnecessary biopsies or prolonged empiric antibiotic therapy. Although our data do not allow us to determine whether earlier use of WB-MRI would have shortened the diagnostic interval or altered clinical outcomes, as imaging was performed after prolonged symptom duration rather than at disease onset, the substantial lesion burden observed at diagnosis illustrates the extent of disease accumulation associated with delayed recognition. These findings support a lower threshold for obtaining WB-MRI once CNO is considered in the differential diagnosis, particularly in children presenting with multifocal bone pain, unexplained limp, or recurrent symptoms not attributable to infection or malignancy. Systematic use of whole-body MRI not only improves early detection, particularly of subclinical spinal lesions, but also informs timely therapeutic escalation and helps prevent complications such as vertebral collapse or limb length discrepancy.

### Limitations

This study has several limitations. Its retrospective, single-institution design and small sample size limit generalizability. Crucially, because this historical cohort inherently captures patients who experienced a prolonged timeline to final diagnosis, WB-MRI was rarely deployed at the immediate onset of initial clinical symptoms. Consequently, our data cannot directly prove that implementing WB-MRI accelerates early clinical remission. Instead, this cohort serves to demonstrate the substantial, asymptomatic structural burden that quietly accumulates across the skeleton when diagnosis is delayed, providing a stark baseline justification for lowering the clinical threshold to perform systematic baseline WB-MRI whenever CNO is suspected. Furthermore, while WB-MRI is highly sensitive for tracking subclinical disease activity, recent landmark studies have demonstrated that asymptomatic, high-signal bone marrow intensities can occasionally be observed in completely healthy pediatric cohorts [21, 22]. Because our study is retrospective, distinguishing normal developmental variants or physiologic marrow conversion from true, low-grade CNO lesions remains an inherent imaging challenge that could lead to an overestimation of lesion burden. Additionally, follow-up imaging was incomplete, preventing systematic assessment of lesion evolution and long-term outcomes. Larger multicenter studies with standardized protocols, incorporating modern pediatric scoring systems to explicitly differentiate physiologic variants from active pathological lesions, and quantitative imaging biomarkers are needed to validate these findings.

## Conclusion

Chronic recurrent multifocal osteomyelitis in children demonstrates a consistent pattern of multifocal metaphyseal marrow edema with frequent clavicular involvement, features that distinguish it from infection and malignancy. Importantly, clinical presentation does not reliably predict disease extent: imaging findings were independent of symptom burden, indicating that clinical examination alone cannot assess true disease burden. Although these findings enhance the accuracy of diagnosis, major gaps persist in surveillance guidelines and long-term management strategies. Prospective multicenter studies with longitudinal follow-up are needed to establish evidence-based guidelines for imaging surveillance frequency, treatment response assessment, and duration of imaging follow-up in remission.

## Data Availability

The datasets generated during and/or analysed during the current study are available from the corresponding author on reasonable request.
